# A robust inorganic binder against corrosion and peel-off stress in electrocatalysis†

**DOI:** 10.1039/d4sc04088k

**Published:** 2024-09-18

**Authors:** Joey Andrew A. Valinton, Meng-Yu Lin, Cheng-Han Tsai, Cheng-Te Tsai, Ming-Jia Chiu, Cheng-chau Chiu, Chun-Hu Chen

**Affiliations:** a Department of Chemistry, National Sun Yat-sen University Kaohsiung 80424 Taiwan chunhu.chen@mail.nsysu.edu.tw ccchiu@mail.nsysu.edu.tw; b Green Hydrogen Research Center, National Sun Yat-sen University Kaohsiung 80424 Taiwan; c Center for Theoretical and Computational Physics, National Sun Yat-sen University Kaohsiung 80424 Taiwan

## Abstract

Electrochemical binders used to immobilize electrocatalysts on electrodes are essential in all fields of electrochemistry. However, conventional organic-based binders like Nafion generally suffer from oxidative decomposition at high potentials on anodic electrodes and have high charge transport resistivity. This work proposes the use of acidic redox-assisted deposition to form cobalt manganese oxyhydroxides (CMOH) as a solid-state inorganic binder. CMOH remains stable under high oxidative currents and ensures catalyst adhesion even under significant peel-off stress as shown by experiments involving the alkaline oxygen evolution reaction (OER) using RuO_2_ as a catalyst immobilized on a rotating disc electrode. While the molecular structure of Nafion decays significantly after 45 minutes under OER conditions at 3.86 V, the CMOH binder is able to support the powder catalysts (RuO_2_ and NiO_*x*_) showing stability around 1000 mA cm^−2^ without significant current decay over 24 hours. The robust catalyst adhesion is a result of the formation of chemical bonds between the electrode and the binder and it can be further improved by increasing the applied loading of CMOH. Unlike Nafion, both the OER activity and the diffusion kinetics are not significantly affected by the CMOH binder. It has also been shown that using CMOH as a binder leads to lower charge transfer resistances *R*_ct_ and higher electrochemical surface areas compared to systems using Nafion. This is partially due to the presence of metal sites in different oxidation states which has been shown to increase intrinsic conductivity, facilitating the charge hopping at the binder/electrocatalyst interface. With this, the present work provides a proof-of-concept for inorganic metal oxides as promising solid-state binders for a wide range of applications in electrochemistry, demonstrating CMOH's outstanding characteristic of strong adhesion to support other highly active but adhesion-weak electrocatalysts.

## Abstract 2

The acidic redox-assisted deposition (ARD) of cobalt manganese oxyhydroxides (CMOH) as a solid-state inorganic binder has been demonstrated. CMOH is stable and highly adhesive under high oxidative currents during OER tests.

## Graphical abstract

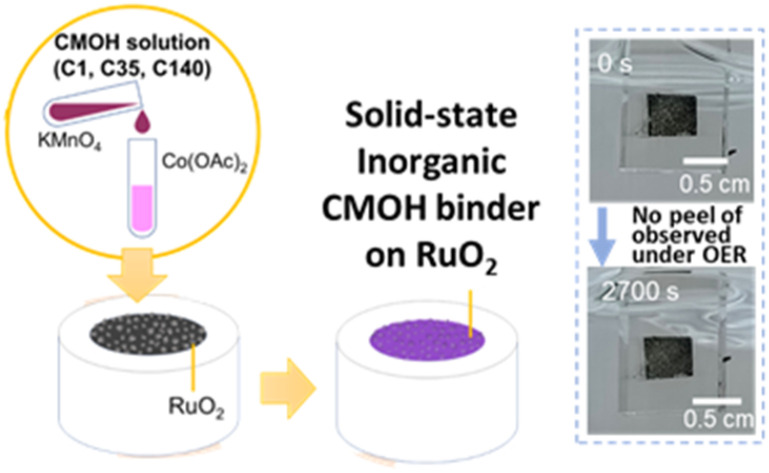

## Introduction

Almost all research setups and devices based on electrochemical processes (*e.g.*, sensors, batteries, supercapacitors, *etc.*) require binders to immobilize electrocatalysts/active materials on the surface of electrodes or current collectors. An ideal binder should provide sufficient adhesion for the tested electrocatalyst and exhibit low electrochemical background noise. Binders based on organic materials (*e.g.*, Nafion, polyvinylidene fluoride, *etc.*) have molecular structures and functionalities that provide a certain level of adhesion for electrocatalysts.^1–5^ However, the insulating nature of organic binders hinders the charge transfer from the surface of current collectors to the active sites of the electrocatalysts. This may eventually result in circumstances where the performance of an electrocatalyst may not be correctly judged when evaluating its electrocatalytic properties.^6,7^

Another issue associated with organic binders is that during electrochemical oxidation processes like the oxygen evolution reaction (OER), the carbon-based backbone of organic binders can be degraded, thus weakening the adhesion of the catalyst, which, in return, affects the reliability of an electrochemical device. Particularly in the case of the OER, operating at high currents leads to the formation of massive amounts of gas bubbles. This, in return, results in strain at the interface between electrocatalysts and electrodes, resulting in possible electrocatalyst peel-off.^1^ Due to these issues, the OER represents one of the harshest electrochemical scenarios for testing binders. Since an increasing number of research studies are focusing on achieving large currents of 500 mA cm^−2^ or higher for water splitting,^8^ new binder materials that can preserve full functionality and structural stability at extremely high oxidative potentials are absolutely essential. Under such conditions, the typical practice of using ionomers like Nafion to bind OER catalysts on electrodes^1,5^ may not be suitable for long-term operation. As reported in a recently published paper by Boettcher *et al.*,^6^ Nafion binder degradation can be observed at a current density of ∼0.5 A cm^−2^ and/or a potential above 2.4 V. This problem is not limited to electrochemical systems in aqueous environments. It is, for instance, equally essential and challenging to develop binders for Li-ion batteries that show decent stability at high oxidative potentials.^9–11^

As long as there is no binder material that is more suitable than the typical organic binders, the most straightforward solution to deal with binder degradation is to apply a higher amount of binders to compensate for it. However, this usually slows down the mass and charge transport. Alternative approaches use carbon materials like carbon black or graphene to immobilize catalysts on a substrate.^8,12,13^ However, the vulnerability of these catalytic systems to degradation under oxidizing environments, resulting in loss of adhesion, remains an unsolved problem.^14,15^

Given the drawback of the binders mentioned above, the concept of “binder-free” electrocatalysts has emerged lately. This typically involves the direct electrochemical deposition of the catalysts onto the electrode surface *via* anodization or electroplating^16–18^ to improve the adhesion of electrocatalysts. Stronger adhesion has been shown to lower the charge transfer resistivity and thus enhances the electrochemical activity.^19,20^ An additional problem faced by many of the earlier studies on binder-free electrocatalysts is that the proposed catalytic systems rely on the interaction of the electrocatalysts with very specific substrates, so the concepts are not universally applicable.

The limited success of the mentioned concepts in immobilizing electrocatalysts indicates that some issues have not been taken into account sufficiently when attempting to design new binders so far. On the one hand, an effective binder should form chemical bonds to reinforce the attachment of the electrocatalyst to the electrode, rather than relying solely on physical adhesion. On the other hand, the binder material should ideally be carbon-free to prevent problems related to oxidative corrosion. To address the latter point, we propose the usage of inorganic binders. The concept of inorganic binders is hardly encountered in the literature as there are several difficulties that need to be overcome when using ordinary inorganic solids like metals, metal oxides, or salts as binders. In general, depositing such solids uniformly on a surface to form a strongly bound layer with atomic-scale thickness is challenging in operation and/or involves high costs.^21,22^ If one wants to develop metal-based binders, one would require metals that are stable enough under extreme electrocatalytic pH conditions, like Au or Pt.^23,24^ However, such metals are often very expensive and are electrochemically too active to be a binder. In contrast, the major problems with ionic salts are that they either tend to dissolve into the aqueous environment or fail to provide sufficiently strong adhesion at relatively low temperatures due to their bonding nature. Similarly, most metal oxides also suffer from weak adhesion unless they are directly grown on the substrate surfaces. In addition, the typically insulating character of metal oxides may also be a problem for electrochemical research.^25,26^

Previously, we developed the acidic redox-assisted deposition (ARD) method to deposit complex oxide coating layers onto the surfaces of metals, ceramics, plastics, *etc.*^27–31^ which has been proven to completely withstand mechanical or electrochemical abrasion. One oxidant often used in ARD is KMnO_4_, which tends to oxidize various substrate surfaces, yielding a directly deposited oxyhydroxide layer. For example, the cobalt manganese oxyhydroxide (CMOH) film through ARD can theoretically possess high-adhesion Mn–O-substrate bonds, which may also occur on various catalyst surfaces.^28^ Both the adhesion and stable structure of CMOH under high potentials, confirmed by *operando* Raman spectroscopy, enhance its electrochemical stability under alkaline electrolyte conditions.^28,29^ The varying oxidation states of the metals within CMOH, due to the redox reaction, are crucial for the electrical conductivity required for charge transport between the substrate and the catalyst.^28,32^ Thus, this work explores whether the new concept of binder materials based on solid-state, inorganic oxides, using CMOH as an example, is possible and compares it to conventional organic binders. Whether the inherent OER activity of CMOH could be an issue that hinders its applicability as a binder, particularly in an experimental set-up evaluating electrocatalysts, is also an issue to be addressed in this study.

Herein, we evaluate whether the properties of ARD-driven oxide coating of CMOH make it a suitable candidate as an inorganic binder. For this, we will focus on evaluating the robustness of electrocatalyst adhesion and resistance against corrosion at elevated oxidative potentials. For a proof-of-concept, we use powdered RuO_2_, as well as NiO_*x*_ as model electrocatalysts and immobilize them with CMOH or with Nafion (as a representative example for organic binders) on an electrode to conduct long-term stability tests. The so-obtained electrodes are then used to perform the OER under alkaline conditions to demonstrate the superiority of the CMOH over organic binders in terms of catalyst adhesion and resistance against oxidative corrosion at elevated potentials. Thus, this work investigates and highlights the role of CMOH as a binder, rather than as an electrocatalyst.

## Results and discussion

### Loading of the inorganic binder and the corresponding properties

**Fig. 1:**
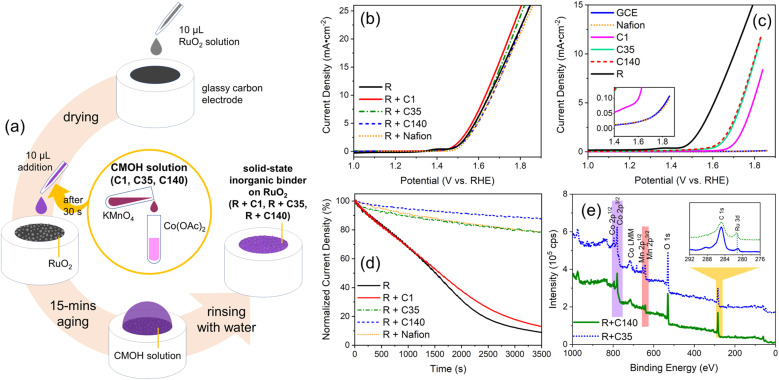
Schematic illustration of the preparation of the RuO_2_ catalyst immobilized by the inorganic solid-state binder CMOH on rotating-disc glassy-carbon electrodes (GCEs) (a) as well as various electrochemical tests and structural characterization studies of RuO_2_ bound with different binders to GCE((b)–(d)) or SiO_2_ wafers (e). (a) To bind the RuO_2_ (denoted as R) drop-cast onto a glassy carbon electrode using CMOH, the binder is deposited onto the electrode surface through the aging of a mixed solution of KMnO_4_ and Co(OAc)_2_ (denoted as CMOH solution, yellow circle) with different concentrations (marked with labels C1, C35, and C140) at room temperature. (b) The linear scan voltammetry (LSV) curves showing the OER activities, under alkaline conditions, of RuO_2_ immobilized by CMOH, prepared using CMOH solutions of varying concentrations. For comparison, the data for binder-free RuO_2_ (R) and Nafion-bound RuO_2_ (R + Nafion) are also shown. (c) The background LSV curves of the (RuO_2_-free) Nafion- and CMOH-only samples. (d) The potentiostatic coulometry curves for the CMOH-bound RuO_2_ samples recorded at 1700 rpm; the vertical axis shows the current densities normalized to the initial current density. The measurements are performed at a potential corresponding to 25 mA cm^−2^ according to (b). (e) The survey XPS spectra of selected CMOH-bound RuO_2_ samples over SiO_2_ wafers; the inset shows the detailed comparison of C 1s and Ru 3d signals.

The typical procedure for applying the CMOH binder to immobilize a tested electrocatalyst is shown in Fig. 1a. In brief, RuO_2_ in powder form is first dispersed in 25% aqueous isopropanol and then drop-cast on the electrochemical substrate, in this case a rotating-disc glassy carbon electrode (GCE), and then covered with a premixed solution containing Co^2+^ and MnO_4_^−^. After 15 minutes of aging, the surface is rinsed with deionized (DI) water to complete the application of CMOH as a solid-state binder.

According to previous studies, the synthesis of CMOH involves the reaction between highly oxidative MnO_4_^−^ and the reductant Co^2+^, as shown in the reaction below:^28,31^

1 9Co_(aq)_^2+^ + 3MnO_4(aq)_^−^ + 14H_2_O_(l)_ → Co_9_Mn_3_O_26_H_13(s)_ + 15H_(aq)_^+^

where Co_9_Mn_3_O_26_H_13(s)_ is the idealized formula of the deposited solid-state species, CMOH. The Co/Mn ratio of the deposit is typically in the range of 3/1 to 2/1.^27^ Previous X-ray absorption spectroscopic characterization of the material has shown that Co is present as Co^3+^ and Mn is present as Mn^4+^.^28^ CMOH exhibits, with a sheet resistance of 13.0 × 10^7^ Ω □^−1^, an electrical conductivity that is two orders of magnitude higher than the corresponding value for the monometal oxides CoO_*x*_ and MnO_*x*_.^28,32^ In addition, these experiments and simulations^28^ have proven that CMOH can form strong chemical bonds with diverse substrates *via* surface oxidation, making CMOH a suitable binder material.

To understand whether and how the applied amount of binder interferes with the intrinsic activity of the electrocatalysts, we varied the concentrations (denoted as *C*) of the CMOH precursor solution and denoted the different solutions as C1, C35, and C140. Samples prepared by combining RuO_2_, abbreviated as R, with the different CMOH precursor solutions are labeled as R + C1, R + C35, and R + C140, respectively. C1 represents a solution with the minimum concentration that is needed to prepare CMOH coatings that have some durability against abrasion and dissolution.^31^ The concentration of C35 is 35 times higher than that of C1. This concentration represents the threshold reported in the literature^28^ that is required to synthesize CMOH samples that begin to show weak OER activity. With C140, we produced a thicker layer of deposition than with C35, as we attempt to study how a thicker solid-state binder layer influences the electrochemical activities.

Fig. 1e displays the X-ray photoelectron spectroscopy (XPS) data for R + C35 and R + C140. In contrast, the spectra of R + C1 are not shown, as it seems to be too thin to give any recognizable signals. The XPS spectra of both R + C35 and R + C140 feature weak but appreciable signals for Co 2p (purple) and Mn 2p (red) which correspond to the characteristic signals of CMOH.^28,29^ The inset of Fig. 1e shows that a weak signal associated with the Ru 3d peak at 281 eV (ref. 33) can be observed. This gives an indication of the thickness of the CMOH binder layer covering the RuO_2_ catalyst. As the sampling depth of XPS is typically in the range of 5 to 10 nm, the thickness of the CMOH layer should be in the same range.^34,35^ This indicates that, even when using the high-concentration CMOH precursor solution C140, the CMOH deposition can still be controlled to form a thin binder layer.

**Fig. 2:**
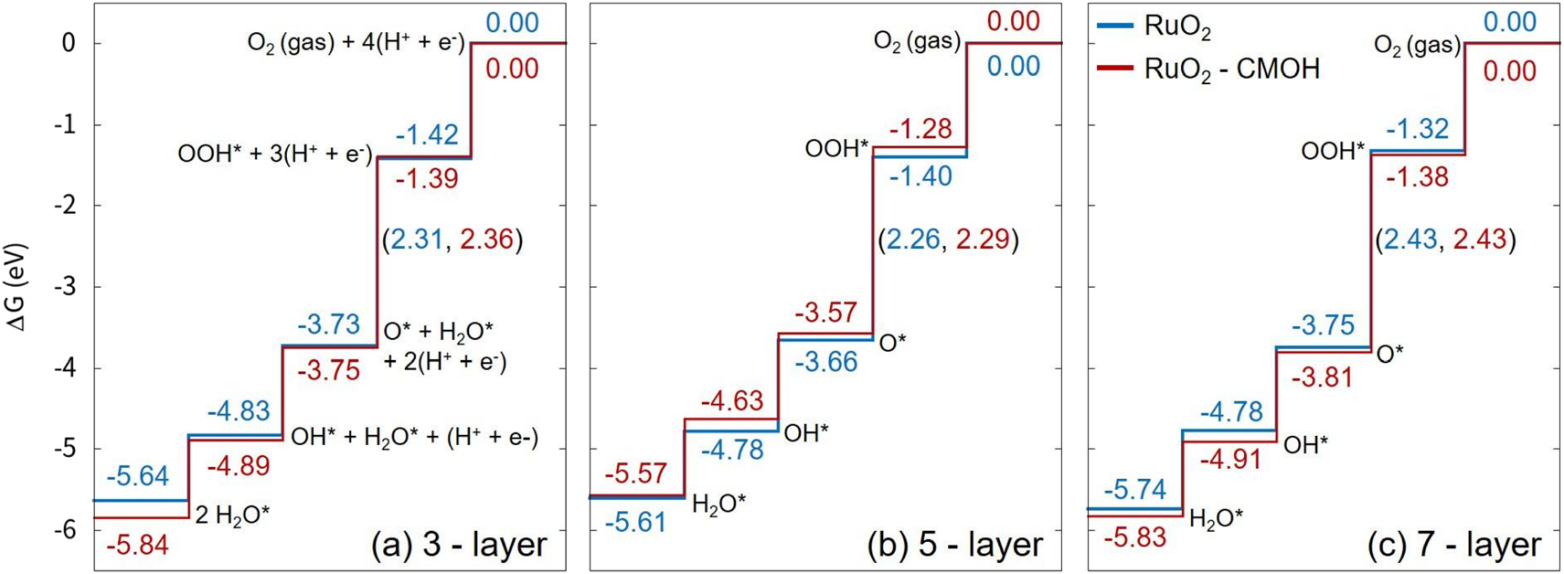
Calculated Gibbs free energy profile for the OER *via* the adsorbate evolving mechanism as reported in ref. 36 on RuO_2_(110) slab models consisting of (a) three, (b) five, and (c) seven RuO_2_ layers, with (red) and without an attached CMOH layer (blue) at the bottom of the slab (all legends in the figure follow the same legend as in figure (c)). The values on the vertical lines are the Gibbs free energies for each surface structure, while the numbers in parenthesis refer to the change in the Gibbs free energy of the corresponding reaction step. All values refer to zero applied potential and are given in eV.

To accurately evaluate the effect of using CMOH as a solid-state binder on the performance of the electrocatalytic system, we have conducted linear scan voltammetry (LSV) measurements under OER conditions while operating the rotating-disc electrode at a relatively high rotation speed of 1700 rpm. Fig. 1b compares the OER activities of Nafion-bound, CMOH-bound, and bare RuO_2_. The difference in the overpotentials (at 10 mA cm^−2^) between the tested samples is relatively small, less than 1.2%. This indicates that neither Nafion nor CMOH alters the intrinsic activity of RuO_2_. It can be further stated that the CMOH depositions obtained with the different precursor solutions C1, C35, and C140 all show behavior similar to that of the organic-binder Nafion. The fact that increasing the concentration of the used CMOH precursor solution has a hardly noticeable effect on the catalytic activity, suggests that even overloading the RuO_2_ catalyst with CMOH does not lead to a significant change in the intrinsic catalytic properties of RuO_2_, nor does it hinder hydroxide ions from accessing the active sites of RuO_2_ effectively.^30^ To confirm that the interaction with CMOH does not noticeably impact the catalytic activity of RuO_2_, we also performed first-principles calculations to evaluate the Gibbs free energies for the OER process on RuO_2_ in the absence and the presence on CMOH following the reaction steps reported for the OER *via* the adsorbate evolving mechanism,^36^ see Fig. 2. Here, we have used RuO_2_ (110) slab models consisting of three to seven layers and added one layer of CMOH at the bottom of the slab to simulate the binder. The computational details and the optimized coordinates of the considered structures are provided in the ESI.† As observed, the presence of the CMOH layer, does not change the potential determining step (PDS), which is the conversion of surface O to surface OOH. Also, the change in the Gibbs free energy for the PDS is, with a value of 0.05 eV or smaller, rather insignificant, indicating that the interaction with CMOH has only a minimal effect on the inherent catalytic activity of RuO_2_.

As shown in Fig. 1c, we evaluated the inherent catalytic activity of the binder material to determine whether it introduces a (too strong) background noise that interferes with the usage of CMOH as a binder in experiments investigating the activity of the bound catalysts. The current density measured for pure Nafion without RuO_2_ is as low as the value for the bare GCE. In contrast, each of the CMOH samples prepared with precursor solutions of different concentrations, particularly C35 and C140, shows some OER activity, which is consistent with earlier literature.^27–29^ In this context, the noticeable background of the samples C35 and C140 may be a concern when they are used with electrocatalysts with weak activity. Thus, when using solid-state binders like CMOH, one should be aware that they are not completely inactive. However, there is no need to overstress this issue as the OER activities of the recently reported electrocatalysts are generally superior to those of the C35 and C140 samples,^37,38^ so one can safely neglect the background noise.

To evaluate the adhesion properties of the CMOH binder, we performed potentiostatic coulometry measurements on the rotating-disc electrodes with CMOH-bound RuO_2_ at a rotation speed of 1700 rpm, see Fig. 1d. The measurements, which lasted one hour, were conducted at potentials corresponding to a current density of 25 mA cm^−2^ according to the LSV data shown in Fig. 1b. Due to experimental uncertainty, the initial current densities recorded in the potentiostatic coulometry measurements vary slightly from 25 mA cm^−2^. Thus, the current densities in Fig. 1d are normalized to the actually measured initial current density for easier comparison (see the ESI† for details). Both R + C1 and binder-free RuO_2_ suffer from a significant decrease in the current density, by 88% and 91% of the initial value, respectively. This is mainly due to RuO_2_ peeling off from the electrode surface, which can be observed in the form of a black powder in the electrolyte. However, we may not completely ignore that there are other minor factors contributing to the observed decrease in the current density, *e.g.*, the accumulation of oxygen bubbles on the electrode surface which cannot be effectively removed even at high rotation speeds.

The fact that the R + C1 sample shows a similar LSV curve as the one recorded for binder-free RuO_2_ shows that the small amount of applied binder material is not enough to withstand the strains caused by bubble formation and the rotation at 1700 rpm leading to the peel-off of the catalyst. In contrast, the R + C35 and R + Nafion samples behave relatively similarly and show about 78% of the initial current density after one hour. For R + C140, even 85–88% of the initial current density could be preserved. Assuming the decrease in current density over time is exclusively due to RuO_2_ peeling off from the electrode, one can state that the adhesive strength of CMOH shows a clear dependence on the concentration of the precursor solution. One may question here, whether this assumption is correct as the higher preserved current density for the R + C140 sample may also be due to the larger amount of applied CMOH, which is also electrocatalytically active. However, this can be safely ruled out as the LSV plots for RuO_2_-free C35 and C140 in Fig. 1c are relatively similar indicating that the inherent activity of the binder material has a negligible effect on the preserved current density. As the R + C140 sample shows the highest stability in terms of OER activity, all samples in the upcoming investigations will be prepared with the precursor solution of concentration C140.

### Effect of increasing binder loading

**Fig. 3:**
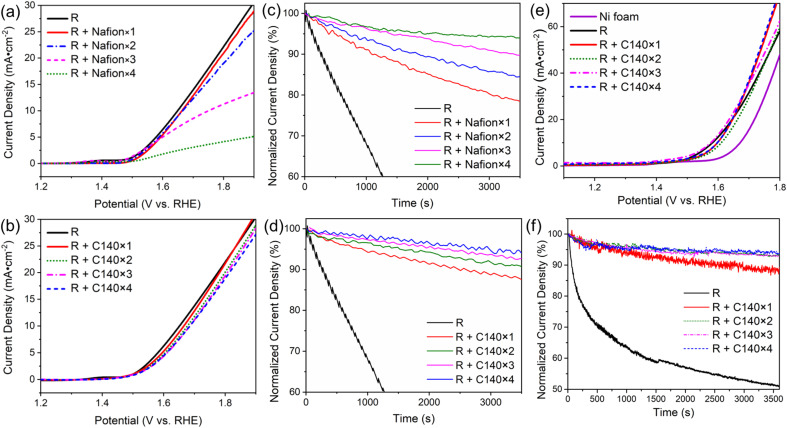
The dependency of the electrochemical behaviors on various loading amounts of binders. Labels of the form “R + Nafion × *n*” or “R + C140 × *n*” refer to systems in which Nafion or CMOH has been deposited n times on RuO_2_ on a rotating-disc GCE ((a)–(d)) or on nickel foam ((e) and (f)). Note that “R + Nafion × 1” and “R + C140 × 1” on the rotating disc GCE represent the samples referred to as “R + Nafion” or “R + C140” in Fig. 1. (a) and (b) The LSV curves measured for electrodes with Nafion- and CMOH-bound RuO_2_, respectively. (c) and (d) The potentiostatic coulometry curves for the Nafion- and CMOH-bound systems measured at 1700 rpm. The measured current densities are normalized to the initial current density. The measurements are performed at a potential corresponding to 25 mA cm^−2^ according to figure (a) and (b), respectively. (e) and (f) The LSV curves and the potentiostatic coulometry curves, respectively, for CMOH-bound RuO_2_ on nickel foam electrodes. The measurements in (f) are performed at potentials corresponding to a current density of 50 mA cm^−2^ according to figure (e). The data for Nafion-bound RuO_2_ are shown in Fig. S2.†

It is generally accepted that one can achieve more stable adhesion of the catalyst by increasing the loading of the binder. However, this usually slows down the overall electrocatalytic process, as reagents and products need to diffuse through the binder to reach the catalyst. Here, we explore how applying multiple layers of binder to immobilize the RuO_2_ catalyst affects the overall electrocatalytic performance. In this case, Nafion or the CMOH precursor solution of concentration C140 has been deposited up to *n* = 4 times on the electrode surface to immobilize the catalyst. The corresponding samples are thus referred to as “R + Nafion × *n*” or “R + C140 × *n*” with *n* being an integer between 1 and 4. Note that R + Nafion×1 and R + C140 × 1 refer to the same set-up previously referred to as “R + Nafion” and “R + C140” when a rotating-disc GCE is used. The LSV plots recorded with Nafion-bound RuO_2_ on a rotating-disc GCE in Fig. 3a show that the generated current beyond the onset potential gradually declines with the loading of the binder. Fig. S12† demonstrates that at 1.8 V, a significant decrease in current density is observed as the number of Nafion binder layers increases from 1 to 4. Notably, adding more than two layers of Nafion results in a dramatic decline in RuO_2_ performance, with up to a 75% decrease in current density. In contrast, the addition of CMOH binder layers retains the RuO_2_ current density more effectively, even with a higher number of layers. On the other hand, the results of potentiostatic coulometry measurements, shown in Fig. 3c, confirm that a higher loading of Nafion indeed leads to a stronger attachment of RuO_2_.^7^ This trade-off between catalytic activity and stability observed for Nafion-bound systems, is less pronounced for systems using CMOH as a binder. The LSV curves for the latter are shown in Fig. 3b: the loading of the binder shows only a negligible influence on the recorded current density. All considered samples have the same OER onset potential. In addition, in the potential range between 1.65 V and 1.85 V, the maximum difference in the measured current density for the samples with different amounts of CMOH never exceeds 3 mA cm^−2^. This indicates that even high loadings of CMOH hardly interfere with the diffusion of the reactant and product species. Similarly, the potentiostatic coulometry results for the CMOH-bound systems in Fig. 3d show that the decay of current density over 1 hour is, across all considered samples, less than 10% of the initial value, and therefore significantly less than the decay observed for the Nafion-bound systems in Fig. 3c. These results highlight an important advantage of CMOH as a binder material over conventional organic materials: it provides increased stability through stronger adhesion of the catalyst without slowing down the diffusion processes.

As mentioned before, a problem with the concept of binder-free catalysts is that they can only be applied to specific substrates. Whether the usage of CMOH and other potential solid-state inorganic binder materials is also limited by this issue is explored in the following. Thus, we have tested whether the CMOH binder can be applied to substrates like the more commonly used porous Ni foam electrodes, which do not feature a flat surface. Compared to glassy carbon, Ni foam is more tolerant to high current densities at high oxidative potentials, which allows us to test the binder materials at higher current densities. The preparation of Ni foam electrodes with CMOH-bound RuO_2_ was conducted similarly to the GCEs used above using the C140 precursor solution. Details of the preparation are described in the ESI.† In this case, all measurements were performed using Fe-free KOH (see the preparation procedure in the ESI†), as Fe tends to adsorb onto the Ni foam electrodes and can lead to an artificial enhancement of the observed OER performance.^39,40^Fig. 3e displays the LSV curves of CMOH-bound RuO_2_ on Ni foam. It appears that the loading of applied CMOH does not affect the measured current density. All samples containing RuO_2_ show very similar overpotentials, which have been determined at a current density of 10 mA cm^−2^. The deviation from the overpotential determined for binder-free RuO_2_ is negligibly small, less than 0.03 V. This indicates that, similar to the situation on GCEs, the CMOH binder does slow down the diffusion processes significantly. The CMOH binder also shows satisfying properties stability-wise, as visible from the potentiostatic coulometry data shown in Fig. 3f. The measurements here are conducted at a potential of around 1.75 V, which corresponds to an initial current density of about 50 mA cm^−2^, roughly twice as high as in the measurements using GCE. The preserved current density after one hour ranges from 88.6% for R + C140 × 1 to 94.0% for R + C140 × 4 showing that the exceptional adhesion of CMOH binders is also maintained on uneven, porous surfaces. In contrast, using Nafion as a binder on Ni foam leads to the same problems observed on GCE. Fig. S2a and b† demonstrate that Nafion, on the one hand, interferes with the diffusion processes, and on the other hand, provides much weaker adhesion of the catalyst to electrode surfaces. According to results from previous studies,^28,29^ CMOH has the potential to be used as a binder material for various other substrates including fluorine-doped tin oxide, carbon felt, and Au-plated silicon oxide.

### Effect of the binder on bubble evolution at high oxidative potential

**Fig. 4:**
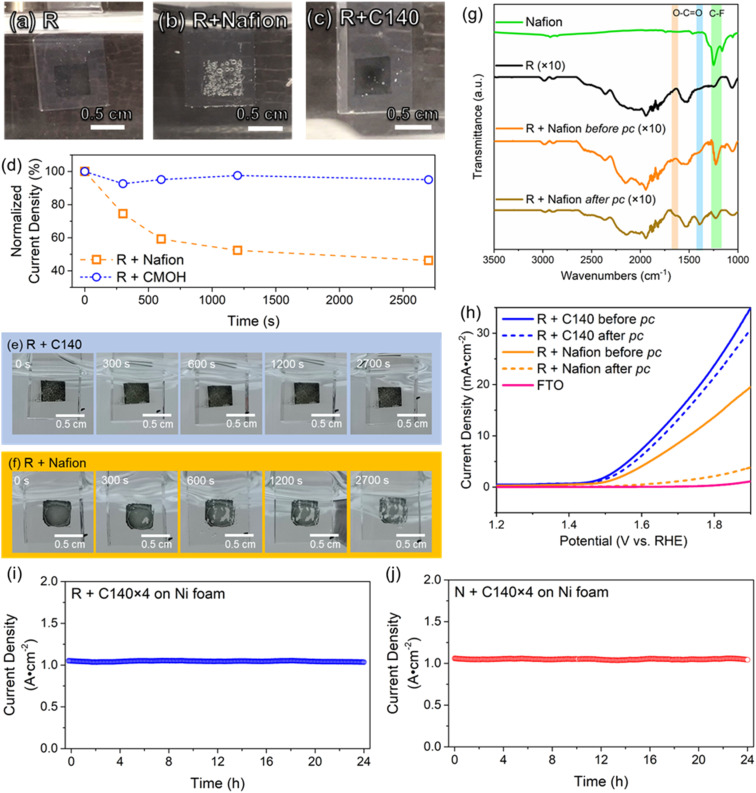
The comparison study of electrocatalytic behaviors and stability between different binders. The bubbling behaviors between different binders under OER conditions for (a) binder-free RuO_2_ (R); (b) Nafion-bound RuO_2_ (R + Nafion); and (c) CMOH-bound RuO_2_ (R + C140). The sizes and local distribution of the as-formed oxygen bubbles can be observed. (d) The peel-off stress tests between the binders under high potential conditions (>3.5 V) of the OER, where the corresponding visual images of the electrode surface are shown in (e) with binder C140 and (f) with binder Nafion. Note that the recorded current densities are normalized to the first initial current densities. (g) The infrared spectra of the electrodes with Nafion-bound RuO_2_ before and after the potentiostatic coulometry (referred to pc) tests shown in (d). The signals of C–F vibrations are highlighted in yellow and the vibrational modes of O–C=O are highlighted in orange and light blue. (h) The LSV results corresponding to the tests shown in (d). The long-term potentiostatic coulometry tests for CMOH-bound RuO_2_ (i) and NiO_*x*_ (j) on nickel foam, showing a current density of around 1000 mA cm^−2^. The constant potential of the long-term potentiostatic coulometry tests for CMOH-bound RuO_2_ is 2.92 V while for CMOH-bound NiO_*x*_, it is 3.18 V.

In all electrocatalytic processes involving the formation of gaseous products, the rapid removal of bubbles is crucial for achieving a faster reaction rate, as the bubbles hinder the transport of reactant species to the electrode. The removal of bubbles can, for instance, be facilitated by increasing the surface hydrophilicity. In the following, we want to visually compare the influence of the binder material on the bubble removal process. For this, we will be using glass coated with fluorine-doped tin oxide (“FTO glass” or simply “FTO”) as electrodes as it is transparent. The details of the preparation of FTO glass electrodes are provided in Section S1.5 of the ESI.†Fig. 4a and c show photographs of FTO electrodes with binder-free and CMOH-bound RuO_2_ referred to as R + C140, respectively, during the OER process. The comparison of bubble sizes on R + C140 and R + Nafion (Fig. 4b and S13†) show that the diameters vary in a range of 0.050–0.073 cm. Nevertheless, a significantly higher bubble coverage on the electrode surface is observed on R + Nafion (80–87% coverage) compared to R + C140 (3–5% coverage). A higher bubble coverage on the electrode surface obstructs catalytic active sites, and thus inhibits the electrochemical activity. The unwanted severe accumulation of oxygen in the form of bubbles in the latter case is most likely due to the hydrophobic perfluoroalkyl backbone of Nafion. On a hydrophobic surface, gas bubbles tend to accumulate and undergo coalescence, leading to the formation of larger bubbles. This large bubble formation could significantly reduce the exposed catalytic sites, thereby potentially diminishing the catalytic performance.^41^ Although the present work does not show a clear difference in the overpotential between Nafion- and CMOH-bound RuO_2_, the slow removal of the bubbles from Nafion-bound catalysts is discussed as the cause for increased overpotentials.^2,41^

As mentioned above, one of the main drawbacks of organic binders is their instability at high oxidative potentials. In the following experiment, we use a potentiostatic coulometry test to visually demonstrate the difference between the performance of Nafion and CMOH under OER conditions at relatively high potentials greater than 3.5 V (also see the details in Fig. S3†). After 45 minutes, the system using CMOH as a binder (R + C140) still retains >90% of the initial current density. In contrast, the current density recorded with the electrodes using Nafion as a binder (R + Nafion) drops to 46% of the initial value. The photographs in Fig. 4e and f (also Fig. S4†) show that the initial appearance of the FTO electrode with CMOH-bound RuO_2_ has not changed significantly upon the potentiostatic coulometry measurement. In contrast, Nafion-bound RuO_2_ has majorly peeled off from the FTO electrode likely due to oxidative corrosion, leading to the degradation of the Nafion layer. To confirm this, Fourier transform infrared (FTIR) spectroscopy has been used to analyze the Nafion layer before and after the potentiostatic coulometry measurement. Fig. 4g shows that after the potentiostatic coulometry measurement, the FTO electrode with Nafion-bound RuO_2_ features peaks at 1646 cm^−1^ and 1391 cm^−1^, which were not observed before the potentiostatic coulometry experiment. These two signals are associated with the symmetric and asymmetric stretching modes of the carboxylate (O–C=O) functional group.^42^ In addition, the decay of the signal intensities of the peaks at 1248 cm^−1^ and 1158 cm^−1^ corresponding to C–F vibrations,^42,43^ shows that the F groups have been removed due to oxidative corrosion. The mentioned changes in the FTIR spectra of Nafion-bound RuO_2_ are consistent with the observations reported for the oxidative degradation of Nafion in the literature.^44,45^ The superiority of the CMOH binder in terms of stability is also visible in the LSV curves measured under OER conditions in Fig. 4h. Here we compare the results for FTO electrodes with Nafion- and CMOH-bound RuO_2_, respectively, before and after the potentiostatic coulometry measurement. While the sample using CMOH as the binder shows comparable current densities, the electrode using Nafion as the binder shows a massive drop.

The high-current stability of the CMOH binder was then evaluated by subjecting CMOH-bound RuO_2_ (R + C140 × 4) and CMOH-bound NiO_*x*_ (N + C140 × 4) on Ni foam to a durability test at around 1000 mA cm^−2^ (see the ESI† for details) in alkaline electrolysis.^8,27^ The constant potentials chosen for the stability test for R + C140 × 4 and N + C140 × 4 are 2.92 V and 3.18 V, respectively, which are already higher than those used with Nafion and other commonly used anion exchange binders^6,46^ NiO_*x*_ was chosen as a representative transition metal catalyst. Fig. 4i displays the potentiostatic coulometry plot for R + C140 × 4 showing nearly no change in electrolyzing current for 24 hours; similarly, the potentiostatic coulometry plot for N + C140 × 4 is shown in Fig. 4j. These results suggest the highly robust adherence of the CMOH binder for electrocatalysts under harsh oxidative conditions induced by high current electrolysis.

### The CMOH binder and the diffusion kinetics

**Fig. 5:**
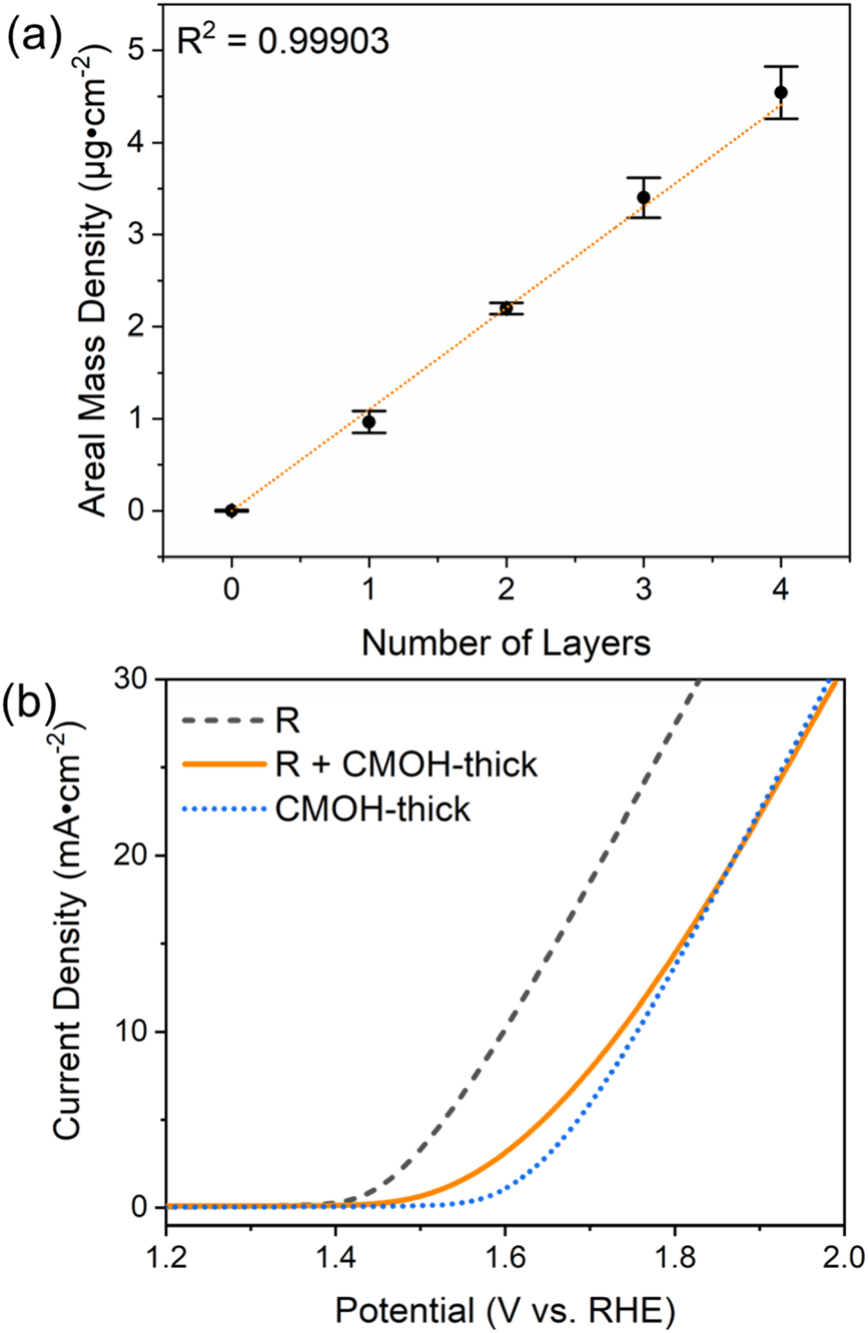
Thickness study of the CMOH binder. (a) The areal mass density measured using a quartz crystal microbalance as a function of the number CMOH layers applied on a quartz cell with a gold electrode using the precursor solution of concentration C140. (b) The LSV curves of RuO_2_ covered with a thick layer of CMOH (R + CMOH-thick) in comparison to those of the binder-free catalyst (R) on GCE and a GCE with only the binder but no catalyst (CMOH-thick).

One of the remarkable features of the CMOH binder is the finding that it seemingly does not hinder the diffusion of the reactant and product species, even if the electrode surface is coated multiple times with the CMOH precursor solution, see Fig. 3b. To confirm this issue, we should first make sure that applying the precursor solution multiple times also leads to a proportional increase in the amount of CMOH actually deposited on the electrode. This issue is important to clarify, as an earlier work from our group has shown that CMOH films formed by immersing the substrate material in the precursor solution at elevated temperatures reach a thickness limit of around 10 nm.^28^ This is related to the interaction of the acetate counter ions in the precursor solutions with the growing CMOH film. In the present study, we used a quartz crystal microbalance (QCM), equipped with a gold-coated SiO_2_ oscillator electrode, to investigate the change in the CMOH areal mass density, *i.e.*, the mass of the binder material per surface area of the electrode, upon applying the precursor solution each time. The detailed experimental procedure is outlined in Section S4.3.1 of the ESI.† Also refer to Fig. S9 of the ESI† for the measured changes in the vibrational frequencies, which are converted into the areal mass densities shown in Fig. 5a. The figure confirms that there is a linear relationship between the areal mass density and the number of times the precursor solution has been applied. With each application of the precursor solution, the areal mass density of CMOH increases by 1.15 μg cm^−2^ on average. The results suggest that there is no limit to the amount of deposited CMOH when the deposition is carried out *via* repeated drop-casting steps. This is likely due to the fact the growth-limiting acetate ions are washed away by rinsing the substrate with water after each drop-casting process. With this issue clarified, we can turn back to the initial question of why the CMOH binder does not hinder the diffusion processes. As argued in Section S4.3.2 of the ESI,† the intuitive assumption that the RuO_2_ catalyst and the electrode surface are completely covered with CMOH is incorrect. Instead, even after depositing four layers of CMOH, only about 26.4 to 44.0% of the surface area of RuO_2_ and the electrode is covered. In other words, large fractions of RuO_2_ remain uncovered, so it is easy to rationalize why the diffusion kinetics are hardly affected.

To provide further evidence for the argument above, we prepared a GCE with RuO_2_ bound by a much thicker layer of CMOH according to the procedure outlined in the ESI.†^28^ The so-prepared binder layer should fully cover the electrode surface and catalyst, and therefore slow down the diffusion processes, which ultimately leads to a smaller observed current density, if the above argument is correct. Fig. 5b shows that RuO_2_ covered with a thick layer of CMOH (R + CMOH-thick) indeed exhibits much lower electrocatalytic activities than binder-free RuO_2_. By comparing the LSV results of R + CMOH-thick to those for a thick layer of CMOH without any RuO_2_, one sees a similar behavior in terms of the recorded current density. We interpret this observation as an indication that the CMOH layer is so thick that the diffusion of hydroxide ions toward RuO_2_, which is necessary for the OER, becomes unlikely.^47,48^ Particularly at elevated potentials, the observed current density is likely dominated by the inherent electrocatalytic activity of the CMOH layer. The similarity to the significantly reduced activity observed for the R + Nafion × 4 sample on the GCE shown in Fig. 3a seems to imply that large loadings of Nafion also hinder the diffusion of OH^−^. Summarizing the insights gained from this part of our work, one can state that the advantage of CMOH is that it does not need to fully cover the catalyst to provide sufficiently strong adhesion. With this, it can be guaranteed that the diffusion of the reactant through the binder material does not become the rate-limiting process.

We further studied the charge transfer resistances *R*_ct_*via* electrochemical impedance spectroscopy (EIS) at the open circuit potential. The detailed procedure is described in the ESI.† This experiment uses nickel foam, which is a common electrode for the large-scale OER. The *R*_ct_ values were derived from the Nyquist plots in Fig. S5† and fitted using an equivalent circuit^49,50^ displayed in Fig. S6.† The corresponding *R*_ct_ values for the electrodes with R + C140 × 1 and R + Nafion × 1 were determined to be 24.11 Ω and 23.52 Ω, respectively, which are slightly higher than the value for binder-free RuO_2_, 20.46 Ω. Quadrupling the CMOH loading yields, the R + C140 × 4 sample, which shows a decrease in *R*_ct_ by 7.7% to 18.88 Ω. In contrast, a four times higher loading of Nafion yields the R + Nafion × 4 sample which features an *R*_ct_ value of 35.48 Ω, showing a +73% increase compared to R + Nafion × 1. We also measured the double-layer capacitance *C*_dl_ to determine the electrochemical surface area (ECSA) for the same electrodes,^29,51^ which is described in the ESI.† It can be seen from Fig. S8† that, compared to the ECSA of binder-free RuO_2_, increasing the amount of the CMOH binder leads to an increase in the ECSA, by up to 2.84 times as observed for R + C140 × 4. In contrast, increasing the amount of Nafion results in a visible decrease of the ECSA.

Previous studies have revealed that metal–oxygen bonds are formed upon growing CMOH on a surface, as KMnO_4_ oxidizes the surface.^28,52^ The growth of CMOH on the substrate and catalyst surfaces should lead to the formation of chemical bonds between the components and contribute to a low interfacial electrical resistance and strong attachment. In addition, the different oxidation states of the Co^III^ and Mn^IV^ cations in CMOH^28,29^ have been shown to provide superior electric conductivities compared to their corresponding single metal oxyhydroxide counterparts, which results in a better overall electrochemical performance.

So far, this study has only evaluated the potential of CMOH as a binder material for aqueous electrochemical systems. Whether it is equally suitable to replace existing binders in systems with non-aqueous electrolytes such as lithium-ion batteries,^53–55^ remains to be studied. However, there are sufficient reasons to be cautiously optimistic. Earlier experiments on lithium-ion batteries using the non-aqueous electrolyte 1-methyl-2-pyrrolidinone showed that adding an outer CMOH layer to the cathode material causes a lowering of the solution resistance by 62% as compared to the control samples without CMOH.^56^ Hence, CMOH may potentially replace the conventional polyvinylidene fluoride (PVDF) binders commonly used in lithium-ion batteries. Given the high stability of CMOH shown in the present study, it may even become the binder material of choice for batteries with high working potentials and large storage capacities.

One issue that may need to be considered when using CMOH as a binder material, is the inherent instability of many metal oxides under acidic conditions. In light of this, CMOH and Nafion may be considered as two binder materials with complementary properties. While the latter with its sulfonate group can facilitate proton transport^42^ and is stable under acidic conditions,^4^ the former is the material of choice for alkaline reaction conditions as demonstrated by this work. Another minor aspect of binder materials, particularly when used in an experimental set-up with reusable electrodes, is how easily the binder material can be removed from the electrode surface. Our tests have shown that this is not an issue to be worried about since CMOH drop-cast on a GCE can be easily removed by polishing with alumina slurry.

## Conclusions

This work provides a proof-of-concept that a solution-based ARD process can be used to deposit a solid state, inorganic binder on various types of common electrodes, regardless of their dimensions and porosity. The CMOH solid-state binder features several advantages over conventional organic binders, including a strong electrocatalyst attachment and robust structural stability at highly oxidative potentials, particularly under alkaline conditions. The results also suggest the potential of CMOH as a good alternative to anion exchange binders. In addition, the CMOH binder has only a negligible impact on mass transport and gives rise to low interfacial electrical resistivity.

Although CMOH has demonstrated some electrocatalytic properties in our earlier work, many other electrocatalysts in the literature exhibit superior electrochemical activity. We believe that advancing the field involves leveraging CMOH's outstanding characteristic of strong adhesion to support other highly active but adhesion-weak electrocatalysts. The design of this research focuses on identifying how CMOH can enhance the stability of other electrocatalysts, rather than continuing to optimize CMOH's activity as done in our previous studies. We believe this solid-state binder concept has a broader impact on the field.

## Author contributions

J. A. A. V. and M.-Y. L. contributed equally. C.-H. C. initiated the concept and provided the main funding for this work. J. A. A. V., M.-Y. L., and C.-H. T. performed the experiments and collected the data. C.-T. T., M.-J. C., and C.-c. C. conducted the computational studies. C.-c. C. provided the funding for the compuational studies of this work. J. A. A. V., M.-Y. L., and C.-H. C. analysed the data. J. A. A. V., M.-Y. L., C.-c. C., and C.-H. C. wrote and edited the paper.

## Conflicts of interest

There are no conflicts to declare.

## Supplementary Material

SC-015-D4SC04088K-s001

SC-015-D4SC04088K-s002

## Data Availability

The data used to support the findings of this study are openly available within the main manuscript and ESI.† The electronic energies and optimized geometries of all considered structures are provided in a separate zip-file in the ESI.†
