# Supplementary material for: A robust inorganic binder against corrosion and peel-off stress in electrocatalysis
Source: Chem Sci. 2024 Sep 18;15(41):16966–76. doi: 10.1039/d4sc04088k (PMC11423651; doi:10.1039/d4sc04088k)
Supplement: SC-015-D4SC04088K-s001 [file SC-015-D4SC04088K-s001.pdf]

Supplementary Information (SI) for Chemical Science.
This journal is © The Royal Society of Chemistry 2024

Supporting Information

Robust Inorganic Binder Against Corrosion and Peel-off Stress in Electrocatalysis

Joey Andrew A. Valinton,[1,2+] Meng-Yu Lin,[1+] Cheng-Han Tsai,[1] Cheng-Te Tsai,[1] Ming-Jia Chiu,[1] Cheng-chau Chiu,[1,2,3*] and Chun-Hu Chen[1,2*]
[1] Department of Chemistry, National Sun Yat-sen University
[2] Green Hydrogen Research Center, National Sun Yat-sen University
[3] Center for Theoretical and Computational Physics, National Sun Yat-sen University
Kaohsiung, Taiwan 80424
*E-mail: chunhu.chen@mail.nsysu.edu.tw

[+] Contributed equally

S1. Preparation of Electrodes with RuO_2 bound by Nafion

S1.1. Preparation of RuO_2 Solution

Commercial RuO_2 powder (anhydrous, 99.9%, Thermo Scientific) was used as the oxygen evolution electrocatalyst. 1.0 mg of RuO_2 was accurately weighted and added into a 10 mL vial. Then, a 3:1 water:isopropyl alcohol mixture is added to obtain a 1.0 mg/mL solution. The vial was then placed into a QSonica Q700 sonicator equipped with a microprobe to treat it with alternating periods of 15 seconds of sonication and 15 seconds of resting, for a total time of 30 minutes.

S1.2. Preparation of the Nafion Solution

A 0.1% (w/v) solution was prepared by diluting 200 μL aliquot of a purchased 5% (w/w) Nafion solution (Sigma-Aldrich) to 10 mL using reagent-grade ethanol.

S1.3. Coating of Rotating Disc Glassy Carbon Electrodes

A rotating disc glassy carbon electrode (GCE, surface area of 0.196 cm^2) was polished with alumina and rinsed with deionized water before use. 10.0 μL of the RuO_2 solution was then drop-cast onto the electrode. Then, the electrode was dried under a halogen lamp. Once the formation of an even coating became visible, a drop, about 10 μL, of the Nafion solution was added and the electrode dried in air. The so prepared electrode is named R + Nafion or R + Nafion×1. Adding 10.0 μL of Nafion solution and drying for n times gave the electrodes with n loadings of Nafion, referred to as R + Nafion×n, with n=2-4.

S1.4. Coating of Ni Foam Substrates

Nickel foam substrates with a geometric surface size of 0.50 cm × 0.50 cm were first immersed in a beaker of water, which was then placed in a sonication bath for 10 minutes. The same procedure is then repeated with ethanol, then acetone, and isopropanol instead of water. The washed foam was then dried in an oven at 70 ºC for six hours, followed by oxygen plasma treatment at 40 W for three minutes to increase the hydrophilicity of the surface. Finally, the Ni foam was soaked in a 1 M HCl solution for ten minutes and then washed with deionized water. After that, 13.0 μL of the RuO_2 solution was dropped onto the Ni foam substrate, which was then dried in air. Then, the same volume of the prepared Nafion solution was added to obtain the electrode labeled

as R + Nafion×1. By adding the 13.0 μL of the Nafion solution n time gave the electrodes with n loadings of Nafion which use the same naming system as applied for the GCE.

S1.5. Coating of FTO Substrates

The surface of FTO glass was washed with acetone, isopropyl alcohol, a concentrated HNO_3 solution of 3.2 M, and finally deionized water. Then, the glass pieces were placed in a box furnace at 500 ºC for two hours, followed by oxygen plasma treatment at 25 W for 25 s. Before adding the RuO_2 solution, Kapton tape was used cover the FTO glass so only a 0.50 cm × 0.50 cm square area of the surface was exposed. 13 μL of the RuO_2 solution was then added onto that exposed area, then the electrodes were then dried in air. After that, 13 μL of the Nafion solution is added to apply the binder.

S2. Preparation of Electrodes with RuO_2 bound by Cobalt Manganese Oxyhydroxide (CMOH) Binder via Acidic Redox-Assisted Deposition

S2.1. Preparation of the CMOH Precursor Solution

7.9 mg (0.032 mmol) of cobalt (II) acetate tetrahydrate [$Co(C_2H_3O_2)_2 \cdot 4H_2O$, Alfa Aesar] were dissolved in 75 mL deionized water to obtain a 0.4 mM solution. In addition, 1.6 mg (0.010 mmol) of potassium permanganate ($KMnO_4$, Showa) was also dissolved in deionized water to get a 0.13 mM solution. The two solutions were then mixed and allowed to react for 30 seconds before applying them to the electrodes for the drop-coating procedure. The concentration of the mixed solution is referred to as C1 in the main text. The CMOH precursor solutions with higher concentrations were prepared similarly but with varying amounts of the added precursors. Solution with a concentration of C35 (35 times C1) was prepared using 0.262 g (1.05 mmol) of $Co(C_2H_3O_2)_2 \cdot 4H_2O$ and 0.055 g (0.34 mmol) of $KMnO_4$, while the solution with C140 (140 times C1) used 1.046 g (4.200 mmol) and 0.221 g (1.40 mmol), respectively. The precursor solution used to prepare electrodes with a thick layer of CMOH (CMOH-thick) was prepared with 1.180 g (4.200 mmol) of cobalt sulfate heptahydrate [$Co(SO_4)_2 \cdot 7H_2O$, Showa] and 0.221 g (1..40 mmol) of $KMnO_4$ in water.

S2.2. Applying CMOH as Binder

Similar to the procedure for the application of Nafion to bind RuO_2 described in Section S1.3-S1.5, the RuO_2 solution is first added onto the GCE, nickel foam, or FTO glass electrodes. Then, the CMOH precursor solution (10.0 μL for GCE, 13.0 μL for Ni foam or FTO glass electrodes) was dropped on the electrodes and aged for 15 minutes. After that, the electrodes were rinsed with deionized water to wash the CMOH precursor solution away and dried by flushing with N_2 gas. The electrodes with RuO_2 bound by CMOH are named R + C1, R + C35, and R + C140 (or R + C140×1), to distinguish the concentration of the precursor solution used for the preparation. To prepare electrodes loaded with n layers of CMOH, the above-described procedure is repeated n times. The so-prepared electrodes are referred to as R + C140×n, with n= 2-4. The GCE with RuO_2 bound by a thick layer of CMOH is prepared with the CMOH-thick precursor solution made of $Co(SO_4)_2 \cdot 7H_2O$ and $KMnO_4$ and is referred to as R + CMOH-thick.

S3. Surface and Material Characterizations

X-ray photoelectron spectroscopy was executed on the CMOH-bound RuO_2 samples on SiO_2 wafers using a PHI 5000 VersaProbe II (Ulvac-Phi, Inc.). Attenuated Total Reflectance Fourier Transform Infrared Spectroscopy of the R + Nafion×1 FTO glass electrodes was performed using a Spectrum Two Instrument (Perkin Elmer) via. The measurements sampled a wavenumber range from 4000 to 450 cm^{-1} with 8 cm^{-1} spectral resolution. In addition, images of binder-free RuO_2 were taken using the FEI Inspect F50 field-emission scanning electron microscope operating at 5 keV.

S4. Electrochemical Characterizations

S4.1. KOH Electrolyte Preparation and Purification

0.1 M KOH solutions are used as the electrolyte solution in electrochemical cells. For the experiments using a GCE, the solution was prepared with KOH pellets (85% purity, Showa) without further purification. For the other experiments using Ni foam or FTO electrodes, the applied 0.1 M KOH solution needed to be Fe-free. The Fe-free solution was prepared as described below [1, 2]:

First, a 1.0 M KOH solution was prepared. Then, nickel (II) hydroxide [$Ni(OH)_2$], which serves as a purifying agent, was prepared by dissolving 3.0 g nickel (II) nitrate (99.9% purity, Sigma-Aldrich) in 4 mL deionized water in a centrifuge tube, followed by the addition of 50 mL 1 M KOH. The mixture was then agitated in an ultrasonic bath for 10 minutes followed by centrifugation at 9000 rpm for 5 minutes. The formed solid precipitate is isolated through decantation. To obtain a Fe-free KOH solution, another 50 mL of 1.0 M KOH was added to the as-prepared, solid $Ni(OH)_2$ in the centrifuge tube to form a dispersion, which was then transferred into a clean 1 L beaker. 450 mL of 1.0 M KOH were further added to the dispersion, which was stirred for 15 minutes and then undisturbed for 1 hour. Afterward, the solid $Ni(OH)_2$ was removed via vacuum filtration using a Buchner funnel with filter paper (Advantec 1, 90 mm). The resulting Fe-free 1.0 M KOH was then diluted to 0.1 M using deionized water.

Figure S1 demonstrates that the potential presence of Fe impurities in the KOH solution does not have significant effects on the measurements using GCE.

S4.2. Electrochemical Cell Setup

The electrochemical tests were conducted using a three-electrode cell composed of the as-prepared GCE, Ni foam, or FTO glass as a working electrode, an Hg/HgO reference electrode, and a Pt wire as the counter electrode. The three-electrode cell was connected to a CHI704E Electrochemical Analyzer (CH Instruments). For the GCE measurements, the rotator (Pine Research) was set to rotate at 1700 rpm.

The potentials reported in this work are all referred to the reversible hydrogen electrode (RHE), E_{RHE}. To convert the potentials measured against the Hg/HgO electrode, $E_{Hg/HgO}$, into E_{RHE}, we use the Nernst equation[3]:

$$E_{RHE} = E_{Hg/HgO} + E^{0}_{Hg/HgO} + 2.303\frac{RT}{F}\left(pH - \frac{1}{2}loglog\,\frac{p_{H2}}{p^{0}}\right) \quad \text{(S1)}$$

Upon assuming that the H_2 pressure p_{H2} is equal to the standard pressure p^0, one gets, after plugging in the values for T = 298.15 K, R = 8.314 J•mol^{-1}·K^{-1}, and F = 96 485.3321 s•A•mol^{-1}, one gets

$$E_{RHE} = E_{Hg/HgO} + E^{0}_{Hg/HgO} + 0.0591\,V{\bullet}pH \quad \text{(S2)}$$

with the standard potential of the Hg/HgO electrode $E^0_{Hg/HgO}$ being 0.098 V.

S4.2.1. Linear Scan Voltammetry

Linear scan voltammetry (LSV) was performed using a scan rate of 5 mV•s^{-1} for measurements using GCE and 50 mV•s^{-1} for experiments with Ni foam electrodes. The potentials shown on the x-axis of the LSV curves are, as mentioned in Section S4.2, referred to RHE In addition, the potentials have been corrected for the ohmic drop, which arises due to the solution resistance[4]:

$$E_{IR-corrected} = E_{RHE} - IR_s \quad \text{(S3)}$$

$E_{IR\text{-}corrected}$ is the corrected potential, I the current measured at a given potential, and R_s the solution resistance as obtained via electrochemical impedance spectroscopy (EIS) at the open circuit potential.

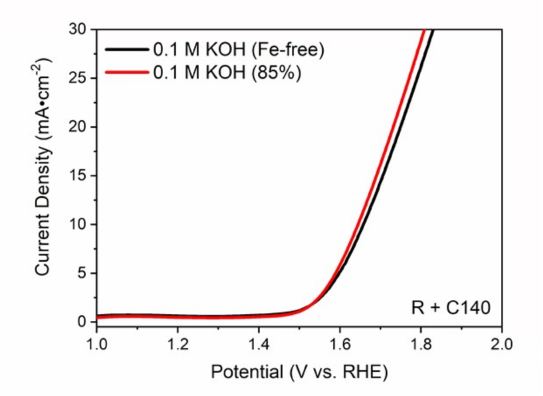

Figure S1. LSV curves recorded with R + C140 on GCE using 85% and Fe-free 0.1 M KOH, respectively, as electrolytes. The electrochemical behavior of the R + C140 electrode shows no significant differences. The changes in the overpotential measured at 10 mA•cm^{-2} is less than 1%.

S4.2.2. Potentiostatic Coulometry

Potentiostatic coulometry measurements were performed at potentials that correspond to a current density of 25 mA•cm^{-1} for GCE and 50 mA•cm^{-2} for Ni foam, respectively, according to the LSV measurements.

In experiments using FTO glass electrodes (Figure 3d), a potential of 3.86 V, corresponding to a current density of 120 mA•cm^{-2}, was applied to study the situation under a highly oxidative environment. The measurements were done in 5-minute intervals followed by rinsing the electrodes to remove all easy-detachable catalysts.

All potentiostatic coulometry curves displayed in this work show the normalized current density which is calculated as:

$$Normalized\ Current\ Density = \frac{current\ density}{initial\ current\ density} \times 100\% \quad (S3)$$

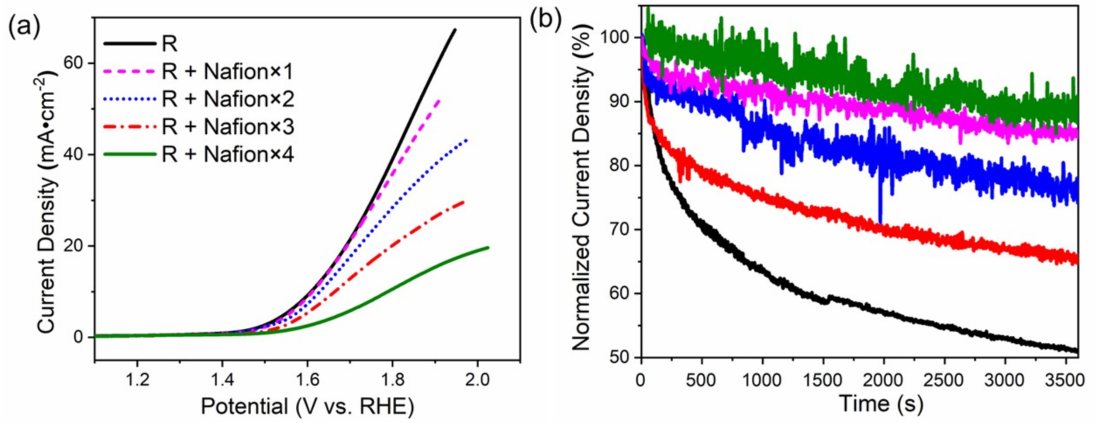

Figure S2. The (a) LSV and (b) potentiostatic coulometry curves of RuO_2 with different loadings of Nafion on a nickel foam electrode. The potentials at which the potentiostatic coulometry measurements have been performed correspond to a current density of 50 mA•cm^{-2} according to the LSV measurements. All measurements here were performed using 0.1 M Fe-free KOH.

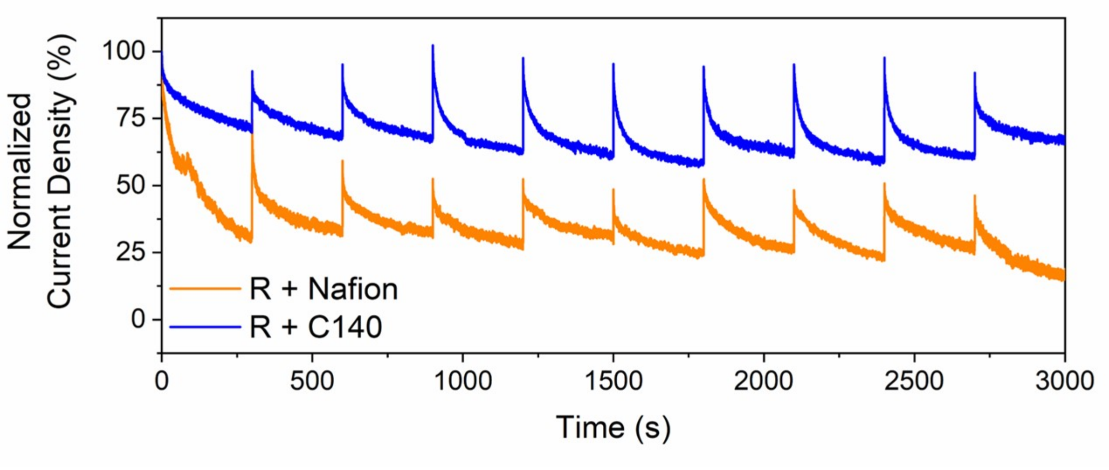

Figure S3. The raw data of the potentiostatic coulometry curves of R + Nafion and R + C140 on FTO electrodes that were used for constructing Figure 3d. At a high potential of 3.86 V, both cases generate too many bubbles on the surface and lead to the delayed kinetics of bubble diffusion away from the surface. This results in the periodic decrease of current density that is not necessarily corresponding to the decay of electrocatalysts. Following the 5- 5-minute interval, the blue curve can restore the current always and is thus recognized to be no decay of the intrinsic activity; but the orange curve cannot restore it and thus actual degradation of electrocatalysts is recognized to be happening. The plot in Fig. 3d was constructed by taking the first set of data points in the beginning of every 5-minute interval from the individual curves. Note that the end of each curve was artificially connected to the start of the next one to act as a guide for the eyes.

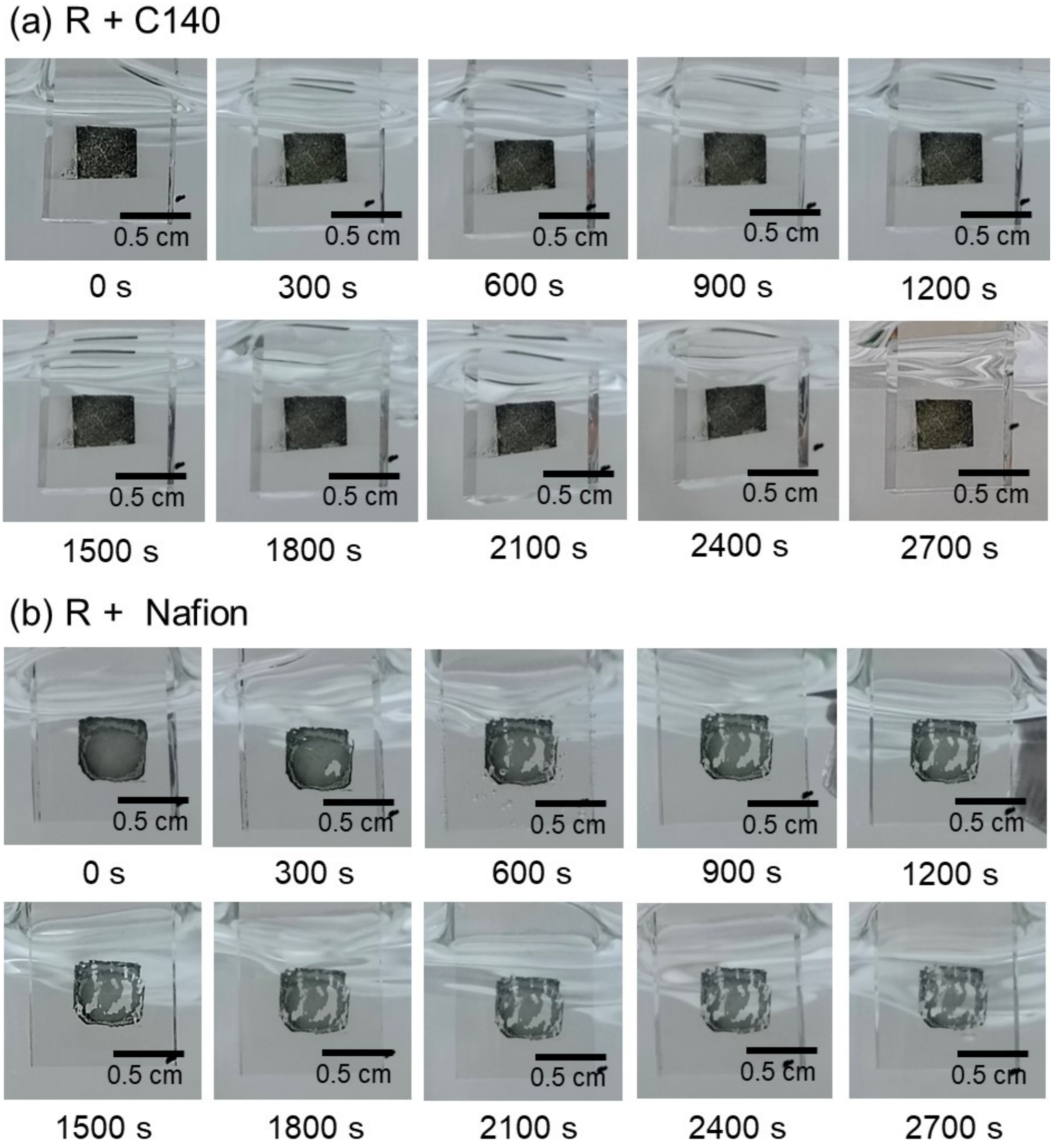

Figure S4. The appearance of the catalyst areas of (a) R + C140 and (b) R + Nafion after each potentiostatic coulometry interval and subsequent rinsing.

S4.2.3. Stability Tests under High Current

C140×4-bound RuO_2 electrode (R+C140×4) was prepared by drop-casting 10 μL of 1 mg•mL^{-1} RuO_2 solution onto an exposed 0.5 cm by 0.5 cm nickel foam. Upon drying, the C140×4 coating procedure (see Section S2.2) was done. The electrochemical setup consists of the prepared working electrode as the anode and a Pt plate as the cathode under a 1 M KOH solution stirred at 400 rpm to induce high mass transport. The whole cell was subjected to potentiostatic coulometry at 2.9 V (without iR compensation) equivalent to the generated current of 1040 mA•cm^{-2}. Regular additions of 2 mL KOH were done per hour to replenish the loss of the solution under high current.

Similarly, NiO_x was also bound to C140×4 (N+C140×4) using the same procedure. NiO_x was prepared by alkaline precipitation of nickel (II) nitrate using ammonium hydroxide through hydrothermal treatment at 80ºC for 24 hours, followed by calcination at 500ºC for 2 hours. The corresponding potential used to generate a current of 1040 mA•cm^{-2} is 3.18 V.

S4.2.4. Observation of Bubbling during OER

To observe the bubbling process, a setup similar to the one used for potentiostatic coulometry was applied. The potential was set to a value of 1.7 V, which is in the OER-active region. The photos of the electrode with the bubbles were taken 2 minutes after the OER process had started.

S4.2.5. Electrochemical Impedance Spectroscopy

In the electrochemical impedance spectroscopy measurements, Nickel foam electrodes with Nafion- and C140-bound RuO_2, respectively, are used as the working electrodes. Measurements are conducted at the open circuit potential with an AC amplitude of 5 mV and frequencies ranging from 1 MHz to 0.1 Hz. Each impedance measurement shown in Figure S3 was fitted to an equivalent circuit as displayed in Figure S4) using the ZSimpWin software to obtain the charge transfer resistance values R_{ct}.

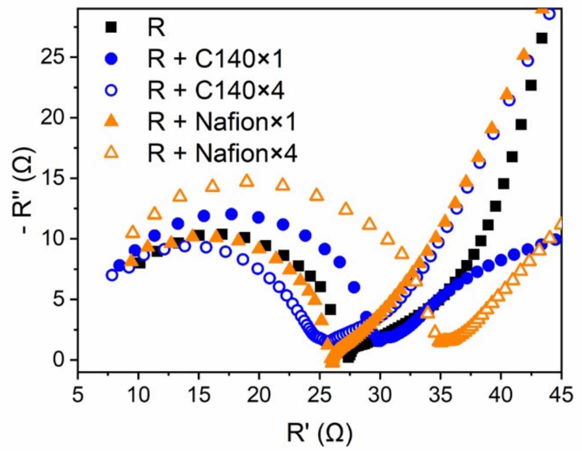

Figure S5. The Nyquist plots obtained from electrochemical impedance spectroscopy measured for nickel foam electrodes showing the effect of the binder material on the charge transfer resistance R_{ct} indicated by the semicircle.

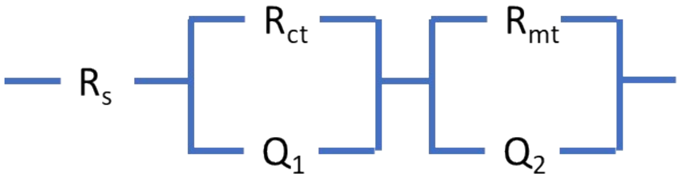

Figure S6. The equivalent circuit used for the fitting of the charge transfer resistance R_{ct}. The other components in the circuit represent the solution resistance R_s, the mass transport resistance R_{mt}, as well as the constant phase elements Q_1 and Q_2 corresponding to non-ideal capacitors.[5]

S4.2.6. Double-Layer Capacitance Measurements

The values for double-layer capacitance C_{dl} were derived from the cyclic voltammetry (CV) curves measured at different scan rates under a non-Faradaic potential range as shown in Figures S5 to S7. The C_{dl} can be determined based on the relationship between the scan rate v and current density j as shown in Figure S8 and the equation below[6]:

$$j = C_{dl}v \qquad \text{(S4)}$$

In this equation, j is the current density measured at the midpoint of the CV curve. Upon obtaining the C_{dl} value for each electrode, the electrochemical surface area (ECSA) could be determined. In this work, we have used the double-layer capacitance for an electrode with binder-free RuO_2, $C_{dl,R}$, as the reference value and defined the ECSA for that system as 1. The ECSA for the other systems "i" were calculated with the following equation[5]:

$$\mathrm{ECSA} = \frac{C_{dl,\,i}}{C_{dl,\,R}} \tag{S5}$$

In this study, “i” can represent R + C140×1, R + C140×4, R + Nafion×1, R + Nafion×4, as well as R.

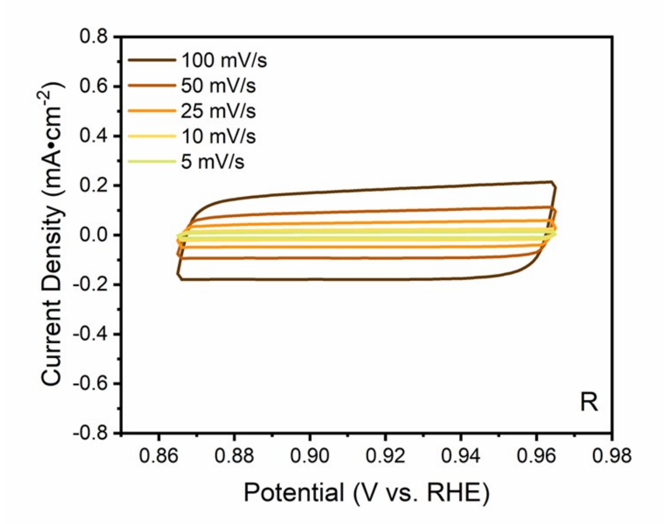

Figure S7. Cyclic voltammetry plots at non-Faradaic potentials using different scan rates for binder-free RuO_2 (R) on Ni-foam.

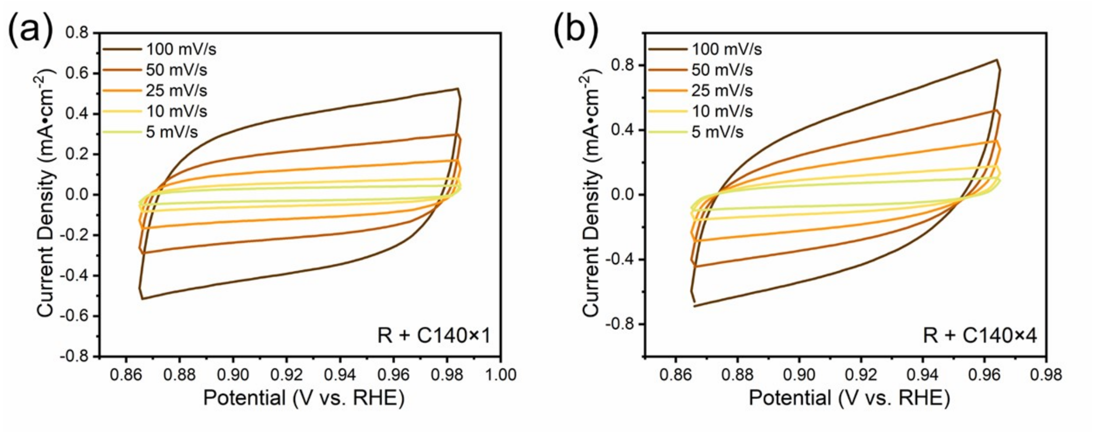

Figure S8. Cyclic voltammetry plots at non-Faradaic potentials using different scan rates for (a) R + C140×1 and (b) R + C140×4 on Ni-foam.

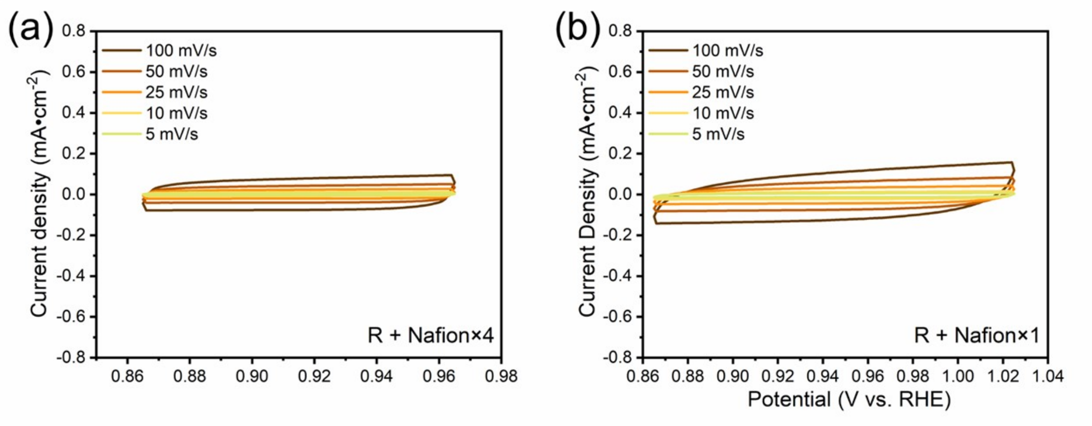

Figure S9. Cyclic voltammetry plots at non-Faradaic potentials using different scan rates for (a) R + Nafion×1 and (b) R + Nafion×4 on Ni-foam.

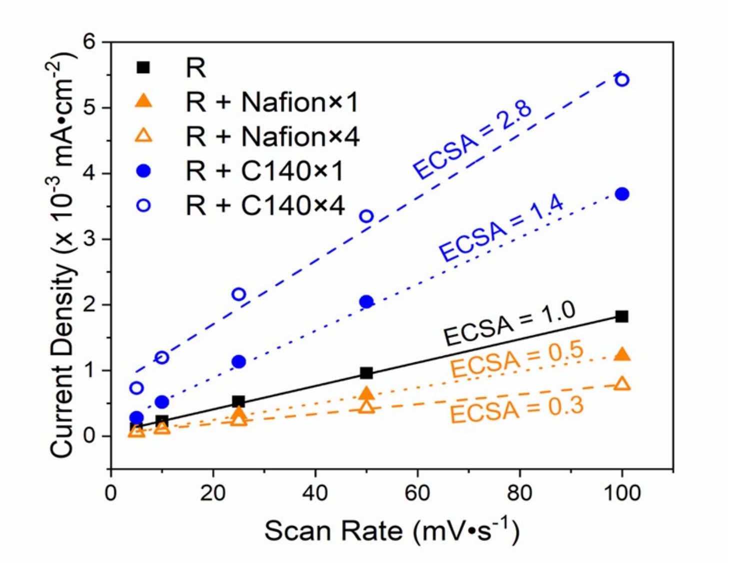

Figure S10. The measured current density as a function of the scan rate derived from the cyclic voltammetry curves measured at non-Faradaic potentials displayed in Figures S4-S7 of the SI to determine the double-layer capacitance C_{dl}. The corresponding ECSA value calculated via eqn S5 of the SI is additionally shown.

S4.3. Quartz Crystal Microbalance

S4.3.1. Setup, Calibration, and Measurement

Quartz Crystal Microbalance (QCM) measurements were performed using a CHI401 Electrochemical Analyzer, where a 7.995 Hz quartz cell with a gold (Au) electrode was used. The idea of the QCM experiment is that the vibrational frequency f of a quartz crystal changes as the areal mass density ρ_A of the material adsorbed on the quartz crystal changes. First, we have obtained the calibration curve showing Δf, the change of the vibrational frequency with respect to the empty quartz crystal, as a function of the areal mass density of the adsorbed material. For this, we have deposited different amounts of Nafion on the quartz crystal, see Figure S9. To do so, we have prepared Nafion solutions of different concentrations c_{Nafion}. Solutions with c_{Nafion} = 1 and 2 mg•mL^{-1} were prepared by diluting 21.7 μL and 43.4 μL, respectively, of a 5% Nafion (Sigma-Aldrich) with ethanol to 1 mL. For a solution with c_{Nafion} = 0.06 mg•mL^{-1}, 60 μL of the prepared 1 mg•mL^{-1} Nafion solution was diluted with ethanol to 1 mL. To prepare the calibrant samples, a defined volume V_{Nafion} of the prepared solutions was dropped onto the quartz crystal. We added 1.0, 1.5, 2.5, and 3.0 μL, respectively of the solution with c_{Nafion} = 1.0 mg•mL^{-1}, 1.0 μL of the solution with c_{Nafion} = 2.0 mg•mL^{-1}, and 3.0 μL of the solution with c_{Nafion} = 0.06 mg•mL^{-1} onto the Au electrode. To prepare a blank sample, we dropped 1.0 μL of reagent-grade ethanol onto the electrode. The area covered by the Nafion coating was regulated by an O-ring with a radius $r_{o\text{-}ring}$ of 0.175 mm. The areal mass densities $\rho_{A,\ Nafion}$ was then calculated using the following formula:

$$\rho_{A,\ Nafion} = \frac{c_{Nafion} V_{Nafion}}{\pi r_{o-ring}^2} \quad \text{(S6)}$$

The equation of the calibration curve obtained in Figure S9 is:

$$\Delta f = (-248.84\ Hz \bullet cm^2 \bullet \mu g^{-1})\rho_{A,\ Nafion} - 1.38\ Hz \quad \text{(S7)}$$

To determine the areal mass density added with each loading of C140, 10 μL of the C140 solution as prepared in Section S2.1 was dropped on the Au electrode of the QCM, so the complete electrode is covered. Then, the solution was then aged for 15 minutes to allow CMOH to form and deposit on the electrode. After that, the electrode

was rinsed three times with deionized water. Water drops remaining on the electrode are removed by gently absorbing them using a Kimwipe. Once the electrode surface is dried the value for Δf value was recorded and converted to areal mass density using eqn. S7. The processes described above are repeated without removing the prior loadings of CMOH to determine the areal mass density of CMOH deposited by multiple loadings.

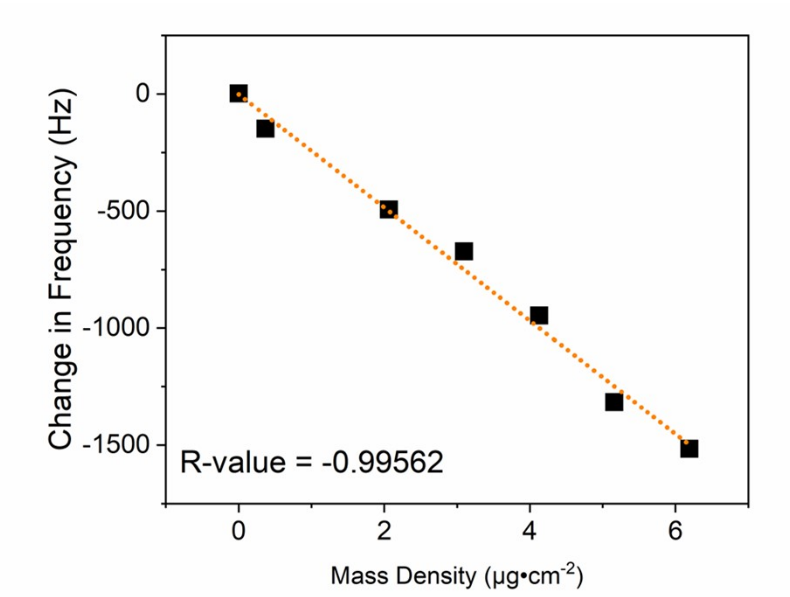

Figure S11. The calibration curve showing the relation between the change in frequency Δf and the areal mass density $\rho_{A,\ Nafion}$ in the quartz crystal microbalance measurements.

S4.3.2. Estimating the Surface Coverage by CMOH

To calculate the ratio of the electrode/RuO_2 surface that is covered by the four deposited layers of CMOH, we used:

$$surface\ coverage = \frac{A_{C140 \times 4}}{A_{RuO2\ +\ electrode}} \tag{S8}$$

where $A_{C140\times4}$ represents the surface area occupied by the CMOH layers, while $A_{RuO2+electrode}$ is the total surface area exposed by the catalyst RuO_2 and the electrode. Assuming that the CMOH layers have a uniform thickness of $l_{C140\times4a}$, one could estimate $A_{C140\times4}$ via the volume of the CMOH layer $V_{C140\times4}$.

$$A_{C140 \times 4} = \frac{V_{C140 \times 4}}{l_{C140 \times 4}} \tag{S9}$$

Earlier studies have indicated that one layer of CMOH has a width between 6 and 10 nm.[7] As we are dealing with four deposited layers of CMOH, we estimate $l_{C140\times4}$ to be four times as large, in the range of 24-40 nm. For the following calculations, we will use $l_{C140\times4}$ = 24 and 40 nm, as lower and upper limit thickness values, respectively to generate the estimated surface coverage range. Meanwhile, $V_{C140\times4}$ can be expressed as:

$$V_{C140 \times 4} = \frac{\rho_{A,C140 \times 4} \bullet A_{RuO2\ +\ electrode}}{\rho_{CMOH}} \tag{S10}$$

where $\rho_{A,\ C140\times4}$ is the areal mass density of the CMOH layers on the electrode obtained from the QCM results for C140×4 in Figure 4a at 4.54×10^{-6} g•cm^{-2}. ρ_{CMOH} stands for the density of CMOH, which is assumed to be equal to the density of CoOOH, 4.30 g•cm^{-3} according to the calculated value taken from the Materials Project database.[8] Plugging eqn. S9 and S10 into eqn. S8 gives:

$$surface\ coverage = \frac{\mu_{C140 \times 4}}{\rho_{CMOH} \cdot l_{C140 \times 4}} \tag{S11}$$

For $l_{C140\times4}$ = 24 nm, the calculated surface coverage is

$$surface\ coverage = \frac{4.54 \times 10^{-6} g \bullet cm^{-2}}{4.30\ g \bullet cm^{-3} \cdot 2.40 \times 10^{-6} cm} = 0.440\ or\ 44.0\% \tag{S12}$$

For $l_{C140\times4}$ = 40 nm, the calculated surface coverage is

$$surface\ coverage = \frac{4.54 \times 10^{-6} g \bullet cm^{-2}}{4.30\ g \bullet cm^{-3} \cdot 4.00 \times 10^{-6} cm} = 0.264\ or\ 26.4\% \tag{S13}$$

Thus, the estimated surface coverage of C140×4 on the electrode is between 26.4 to 44.0%,

S4.3.3. Quantitative impacts of binders on catalytic performance and bubble formation

The change in the current density is observed as the binder amount increases (Figure 3a and 3b) for both Nafion and CMOH applied on the RuO_2 catalyst. Based on those results, we compared the change of current density at 1.8 V as the layer thickness of each binder increases.

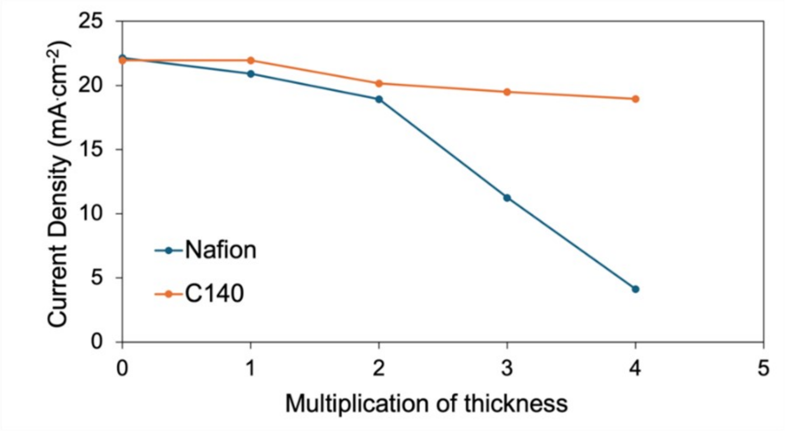

Figure S12. *The change in current density at 1.8 V as the thickness of the Nafion and C140 binder increases.*

We also quantitatively measured and compared bubble formation and size on RuO_2 electrodes with Nafion and C140 binders during the OER test (Figures 4b and 4c). A higher accumulation of bubbles is observed on the RuO_2 with Nafion binder, with bubble sizes ranging from 0.050–0.073 cm compared to 0.048–0.058 cm for R + C140, as shown in Figure S13. The photographs were taken during the OER test at 20, 25, and 35 seconds, and the images were processed using ImageJ to determine bubble sizes.

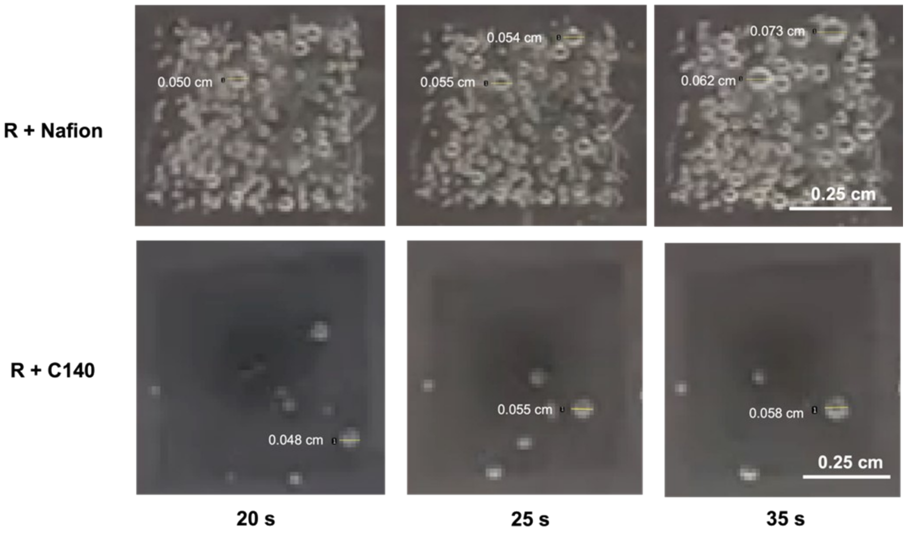

Figure S13. *Photographs showing the change of size of the selected big bubbles with a duration time of 20, 25, and 35 s during the electrolysis of RuO_2 that contains Nafion and C140 binder.*

S.3. Computational Methods

The first-principles calculation reported in this work are spin-polarized, periodic Density Functional Theory (DFT) calculations using the Vienna Ab initio Simulation Package (VASP), version 6.1.2.[9-13] The electronic structures are computed with Perdew, Burke, and Ernzerhof-type (PBE) exchange-correlation (xc) functional.[14,15] While the core electrons are treated with the projector augmented wave (PAW) method,[16, 17] the valence electrons wave function are explicitly described with a plane-wave basis with 450 eV cutoff energy. In addition, Gaussian smearing with a smearing width of 0.05 eV is used. To account for the inaccuracies arising from the self-interaction error in (semi-)local xc functionals, the DFT+U approach[18] has been applied on the d-orbitals of the Co and Mn. The corresponding U-J parameters are 3.52 eV and 3.90 eV, respectively, as take from the literature.[19, 20] The self-consistent field (SCF) calculation of the electronic energies is considered as converged if the energy difference between two iterations is less than 10^{-6} eV. The convergence criteria for the geometry optimization in which all atoms are relaxed require the forces on each atom to be less than 0.02 eV/Å.

The RuO_2 catalyst is modeled by a $RuO_2(110)$ slab model. The simulation cells with the optimized cell parameters a = 6.12 Å and b = 6.33 Å are 2×2 supercells of the orthorhombic surface structure, consisting of three, five, or seven layers of RuO_2 (Figure S14). Each periodically repeated unit of a RuO_2 layer corresponds to a sum formula of Ru_4O_8. To avoid unphysical interactions between the RuO_2 slab and its period image, each slab model is separated by a vacuum space of at least 15 Å. To model the CMOH binder, we have, based on the geometry of Cobalt oxyhydroxide (CoOOH), prepared a 2D structure of 6-fold coordinated CoO_6 and MnO_6 with a local D_{3d} symmetry. As visible in Figure S14b, the structure can be understood as edge-sharing, distorted octahedrons. H-atom are added to form hydroxyl groups and to tune the oxidation state of the metal centers. The sum formula for the CMOH model is $Co_3MnO_5(OH)_3$, which corresponds to the experimentally determined Co/Mn ratio between 3/1 and 2/1 [21] and oxidation states of 3+ and 4+ for Co and Mn, respectively. [22] As illustrated in Figure S14c, the CMOH layer is directly attached to the bottom of the RuO_2 slab to simulate the effect of the binder on the catalyst. Although there is lattice mismatch between CMOH and RuO_2, the cell parameters have not been adjusted for the calculations. This is to account for the fact that when the binder is applied in the experiment it is deposited on the catalyst (and the substrate), so the RuO_2 structure

should dictate unit cell parameters of the catalyst-binder system. The Brillouin zones of the structures above are sampled with a 3×3×1, Monkhorst-Pack-type k-point grid.[23] To simulate the OER process, the intermediates at place on the exposed metal sites of the RuO_2(110) surface as exemplarily shown in Figure 2. The energy reference for gas phase species is obtained by placing the molecule in a 20 Å×20 Å×20 Å simulation cell, while sampling the reciprocal space at the Γ-point only.

Numerical normal mode analyses within the harmonic approximation has been performed for considered geometries. However, to reduce the computational costs, only the atoms of the adsorbed surface species have been considered for the normal mode analysis. The Gibbs free energies reported in this work all refer to reaction conditions of 0 pH, 0 V applied potential, and a temperature of 298 K and have all been obtained using the concept of the computational hydrogen electrode, which exploits the fact that a H^+ + e^- pair is having the same Gibbs free energy of 1/2 H_2 molecule at 1 bar. [24, 25]

The electronic energies and optimized geometries of all considered structures are provided in a separate zip-file in the SI.

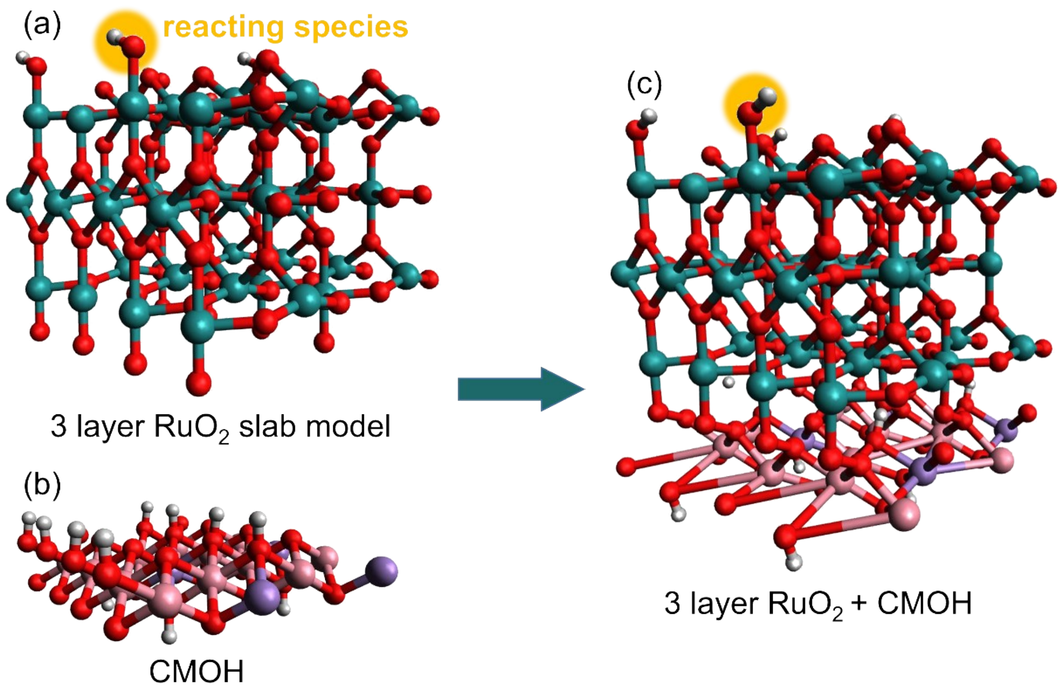

Figure S14. Schematic representation of (a) a RuO_2(110) slab model with three layers, (b) the CMOH model, and (c) a RuO_2 slab model with CMOH attached to it.

References:

1. L. Trotochaud, S. L. Young, J. K. Ranney and S. W. Boettcher, *J Am Chem Soc*, 2014, **136**, 6744-6753.
2. R. A. Márquez, K. Kawashima, Y. J. Son, G. Castelino, N. Miller, L. A. Smith, C. E. Chukwuneke and C. B. Mullins, *ACS Energy Letters*, 2023, **8**, 1141-1146.
3. A. J. Bard and L. R. Faulkner, Electrochemical Methods: Fundamentals and Applications, 2nd Ed. Wiley: New York, 2000
4. W. Zheng, *ACS Energy Letters*, 2023, **8**, 1952-1958.
5. J. A. A. Valinton, M.-C. Chung and C.-H. Chen, *The Journal of Physical Chemistry Letters*, 2022, **13**, 4200-4206.
6. C. C. L. McCrory, S. Jung, J. C. Peters and T. F. Jaramillo, *Journal of the American Chemical Society*, 2013, **135**, 16977-16987.
7. R.-H. Jhang, C.-Y. Yang, M.-C. Shih, J.-Q. Ho, Y.-T. Tsai and C.-H. Chen, *Journal of Materials Chemistry A*, 2018, **6**, 17915-17928.
8. A. Merchant, S. Batzner, S. S. Schoenholz, M. Aykol, G. Cheon and E. D. Cubuk, *Nature*, 2023, **624**, 80-85.
9. G. Kresse and J. Hafner, *Physical Review B*, 1993, **47**, 558-561.
10. G. Kresse and J. Hafner, *Physical Review B*, 1994, **49**, 14251-14269.
11. G. Kresse and J. Furthmüller, *Computational Materials Science*, 1996, **6**, 15-50.
12. G. Kresse and J. Furthmüller, *Physical Review B*, 1996, **54**, 11169-11186.
13. G. Kresse and J. Hafner, *Journal of Physics: Condensed Matter*, 1994, **6**, 8245.
14. J. P. Perdew, K. Burke and M. Ernzerhof, *Physical Review Letters*, 1996, **77**, 3865-3868.
15. J. P. Perdew, K. Burke and M. Ernzerhof, *Physical Review Letters*, 1997, **78**, 1396-1396.
16. P. E. Blöchl, *Physical Review B*, 1994, **50**, 17953-17979.
17. G. Kresse and D. Joubert, *Physical Review B*, 1999, **59**, 1758-1775.
18. S. L. Dudarev, G. A. Botton, S. Y. Savrasov, C. J. Humphreys and A. P. Sutton, *Physical Review B*, 1998, **57**, 1505-1509.
19. M. Bajdich, M. García-Mota, A. Vojvodic, J. K. Nørskov and A. T. Bell, *Journal of the American Chemical Society*, 2013, **135**, 13521-13530.
20. D. A. Kitchaev, H. Peng, Y. Liu, J. Sun, J. P. Perdew and G. Ceder, *Physical Review B*, 2016, **93**, 045132.
21. Y. Devi, P.-J. Huang, W.-T. Chen, R.-H. Jhang and C.-H. Chen, *ACS Applied Materials & Interfaces*, 2023, **15**, 9231-9239.
22. R.-H. Jhang, C.-Y. Yang, M.-C. Shih, J.-Q. Ho, Y.-T. Tsai and C.-H. Chen, *Journal of Materials Chemistry A*, 2018, **6**, 17915-17928.
23. H. J. Monkhorst and J. D. Pack, *Physical Review B*, 1976, **13**, 5188-5192.
24. E. Skúlason, T. Bligaard, S. Gudmundsdóttir, F. Studt, J. Rossmeisl, F. Abild-Pedersen, T. Vegge, H. Jónsson and J. K. Nørskov, *Physical Chemistry Chemical Physics*, 2012, **14**, 1235-1245.
25. J. K. Nørskov, J. Rossmeisl, A. Logadottir, L. Lindqvist, J. R. Kitchin, T. Bligaard and H. Jónsson, *The Journal of Physical Chemistry B*, 2004, **108**, 17886-17892.
